# Neuroprotection by inhibiting the c-Jun N-terminal kinase pathway after cerebral ischemia occurs independently of interleukin-6 and keratinocyte-derived chemokine (KC/CXCL1) secretion

**DOI:** 10.1186/1742-2094-9-76

**Published:** 2012-04-25

**Authors:** Corinne Benakis, Anne Vaslin, Christian Pasquali, Lorenz Hirt

**Affiliations:** 1 Department of Clinical Neuroscience, University Hospital of Lausanne, (CHUV), BH 07, Lausanne 1011, Switzerland; 2 Department of Clinical Neuroscience, University Hospital of Lausanne, Xigen SA, Epalinges 1066, Switzerland

**Keywords:** c-Jun N-terminal kinase, Inflammation, Interleukin-6, Keratinocyte-derived chemokine, Middle cerebral artery occlusion, Neuroprotection

## Abstract

**Background:**

Cerebral ischemia is associated with the activation of glial cells, infiltration of leukocytes and an increase in inflammatory mediators in the ischemic brain and systemic circulation. How this inflammatory response influences lesion size and neurological outcome remains unclear. D-JNKI1, an inhibitor of the c-Jun N-terminal kinase pathway, is strongly neuroprotective in animal models of stroke. Intriguingly, the protection mediated by D-JNKI1 is high even with intravenous administration at very low doses with undetectable drug levels in the brain, pointing to a systemic mode of action, perhaps on inflammation.

**Findings:**

We evaluated whether D-JNKI1, administered intravenously 3 h after the onset of middle cerebral artery occlusion (MCAO), modulates secretion of the inflammatory mediators interleukin-6 and keratinocyte-derived chemokine in the plasma and from the spleen and brain at several time points after MCAO. We found an early release of both mediators in the systemic circulation followed by an increase in the brain and went on to show a later systemic increase in vehicle-treated mice. Release of interleukin-6 and keratinocyte-derived chemokine from the spleen of mice with MCAO was not significantly different from sham mice. Interestingly, the secretion of these inflammatory mediators was not altered in the systemic circulation or brain after successful neuroprotection with D-JNKI1.

**Conclusions:**

We demonstrate that neuroprotection with D-JNKI1 after experimental cerebral ischemia is independent of systemic and brain release of interleukin-6 and keratinocyte-derived chemokine. Furthermore, our findings suggest that the early systemic release of interleukin-6 and keratinocyte-derived chemokine may not necessarily predict an unfavorable outcome in this model.

## Findings

### Introduction

An increase in cytokines and chemokines in the central nervous system and in the systemic circulation has been reported in experimental cerebral ischemia as well as in patients with acute stroke [1,2]. Several studies showed an association between inflammatory mediators and brain damage, stroke progression and severity [3,4].

The intracellular c-Jun NH_2_-terminal kinase (JNK) pathway is regulated in response to cerebral ischemia, leading to the activation of apoptotic and inflammatory transcription factors [5,6]. D-JNKI1 is a selective JNK inhibitor [7,8] that protects neuronal cultures against excitotoxicity and induces a high degree of neuroprotection in several models of cerebral ischemia, both *in vitro* and *in vivo*[9,10]. The protection can be achieved by intra-cerebroventricular injection and also by intravenous administration at very low doses with drug levels below detection in the brain [11,12], suggesting a systemic mode of action such as on inflammation. Indeed, others showed that D-JNKI1 displays anti-inflammatory properties in models of hemorrhagic shock and resuscitation [13] and endotoxin-induced uveitis [14]. We have previously shown that microglial activation after middle cerebral artery occlusion (MCAO) is not affected by D-JNKI1 neuroprotection *in vivo* while *in vitro* D-JNKI1 has an obvious effect [12]. Here, we investigated the influence of this potent neuroprotectant on inflammatory mediators. Based on recent findings that the cytokine interleukin-6 (IL-6) and the mouse ortholog of IL-8, keratinocyte-derived chemokine (KC/CXCL1), displayed the most significant changes in release following cerebral ischemia [15] and knowing that JNK regulates their transcription [16], we investigated whether part of the neuroprotective effect of D-JNKI1 results from the modulation of systemic and brain secretion of IL-6 and KC following cerebral ischemia in mice.

## Methods

Focal ischemia was induced in outbred male Crl:CD1/ICR mice (25 to 35 g) using the filament method as previously described [11,12]. All procedures were in accordance with the Swiss Federal Law on Animal Welfare and were approved by the Swiss Cantonal Veterinary Office. Briefly, the left middle cerebral artery was occluded for 30 min and the filament was withdrawn to allow reperfusion. Sham animals underwent the procedure without arterial occlusion. Cerebral blood flow and temperature were recorded. Randomly, a single dose of vehicle solution (0.85% sodium chloride) or D-JNKI1 (0.1 mg/kg, NeoMPS, obtained from Xigen SA, Epalinges, Switzerland) was injected intravenously 3 h after ischemia onset. Mice were killed at different time points after MCAO.

Plasma was obtained from cardiac blood samples (1/16 v/v of 4% sodium citrate) by centrifugation (15 min at 2500 rpm, 4°C). Mice were transcardially perfused with PBS. To evaluate the cytokines released by the tissue rather than those present intracellularly, we assayed the culture medium of brain and spleen samples after an overnight incubation [17,18]. Coronal brain slices 2 mm thick and a segment of the spleen 5 mm thick were incubated in wells containing 1.5 mL of DMEM medium (Dulbecco’s Modified Eagle Medium 1X, 4.5 g/L glucose, L-glutamine, GIBCO, UK), 10% horse serum (Oxoid Ltd., Basingstoke, UK) supplemented with 11.0 mg/mL sodium pyruvate (100 mM, Sigma, USA) and 10 mL/L penicillin:streptomycin (Sigma, USA) for 20 h at 37°C in humidified air with 5% CO_2_. Control mice were used to distinguish between the release due to MCAO and that resulting from tissue preparation and culture. The medium was centrifuged (5 min at 12,000 rpm, room temperature). The supernatants were frozen at −80°C. Protein concentrations were determined by Bradford assay.

To assess cytokine levels in brain homogenates, the slice adjacent to the one tested for brain cytokine release was directly frozen in liquid nitrogen and conserved at −80°C for further analysis. Frozen sections were cut again into 20 μm slices using a cryostat and the ipsilateral and contralateral hemispheres were separated and collected. Slices were homogenized in buffer (20 mM tris(hydroxymethyl)aminomethane-acetate, pH 7.0; 0.27 M sucrose; 1 mM ethylenediaminetetraacetic acid; 1 mM ethyleneglycoltetraacetic acid; 50 mM sodium fluoride; 10 mM beta-glycerophosphate; 5 mM sodium pyrophosphate; 1 mM sodium vanadate; 1% Triton X) containing protease inhibitors. After centrifugation (15 min at 10,000 rpm, 4°C), supernatants were collected and protein concentrations calculated according to the Bradford method.

An ELISA was performed using half the quantities recommended by the manufacturer for the detection of mouse IL-6 (OptEIA TM Set, BD Biosciences, San Diego, CA) and KC/CXCL1 (DuoSet ELISA Development System, Abingdon, UK) in plasma, supernatants from incubated brains and spleens, and supernatants from brain homogenates. The optical density was measured at 450 nm.

Immunohistochemical labeling and double immunofluorescence of brain sections were performed according to previously published data [12] using the following antibodies: mouse anti-Neuronal Nuclei (NeuN, 1/500, Millipore), mouse anti-Glial Fibrillary Acidic Protein (GFAP, 1/500, Millipore), rat anti-CD11b (Mac-1, 1/100, AbD Serotec, UK), rabbit polyclonal anti-IL-6 antibody (1/200, ab6672, Abcam, Cambridge, UK), anti-growth-related protein alpha antibody against chemokine KC/CXCL1 (GRO-alpha, 1/100, ab86436, Abcam, Cambridge, UK). Images were acquired with the Aviovision v3.1 software using a Zeiss Axiovision microscope.

Results were expressed as mean ± standard error of the mean (SEM). Cytokine concentration data were square root-transformed to bring distributions closer to normal and reduce variance inequalities before statistical analysis. Comparisons between three or more groups were performed using one- or two-way analysis of variance. For post-hoc comparisons, a Brown-Forsythe test for equality of variances between groups was performed, followed by Tukey’s comparison of means when variances were equal or a Games-Howell test when variances were unequal. Paired statistical tests were used to compare cytokine concentration in the ipsilateral versus the contralateral hemisphere at each time point for both the MCAO+vehicle and the MCAO+D-JNKI1 mice (JMP 9.0.0 and IBM SPSS statistics 19.0.0). *P* <0.05 was considered significant.

## **Results**

**Figure 1:**
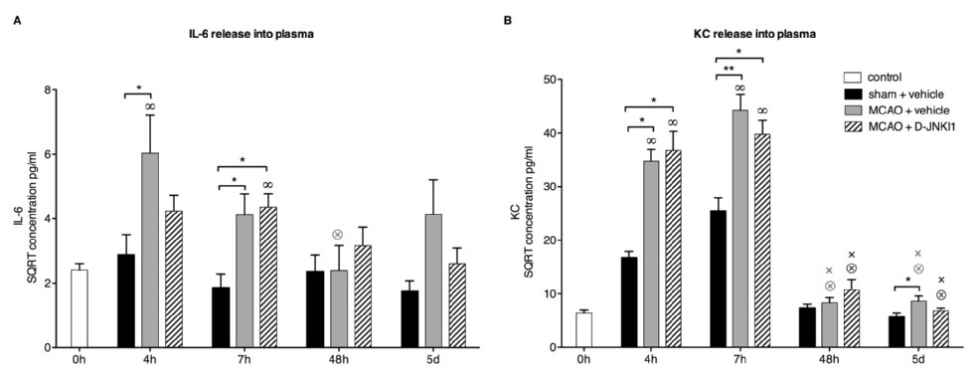
**Two peaks of interleukin-6 and keratinocyte-derived chemokine release into the plasma after middle cerebral artery occlusion.** Square root-transformed (SQRT) IL-6 and KC plasma concentrations at different time points in control mice, sham and MCAO mice treated with vehicle or D-JNKI1. (**A**) In sham mice, IL-6 did not differ from control mice at any time point. Plasma IL-6 was induced 4 h and 7 h after MCAO+vehicle at a significantly higher level than sham mice. At 24 h (data not shown) and 48 h, IL-6 concentrations in MCAO+vehicle mice returned to basal level and increased again 5 d after the occlusion (sham versus MCAO+vehicle: *P* = 0.054). The plasma IL-6 concentration in MCAO+D-JNKI1 mice was slightly reduced at 4 h and 5 d but not statistically different from MCAO+vehicle mice (4 h: MCAO+vehicle versus MCAO+D-JNKI1: *P* = 0.26; 5 d: MCAO+vehicle versus MCAO+D-JNKI1: *P* = 0.27). Seven hours after MCAO, IL-6 concentration in D-JNKI1-treated mice was significantly higher compared with sham mice. (**B**) Plasma KC was induced early in MCAO+vehicle mice compared to sham mice and decreased from 24 h (data not shown) onwards. Five days after MCAO, KC concentration was again significantly higher than in sham mice (sham versus MCAO+vehicle: *P* = 0.03). KC concentration in MCAO+D-JNKI1 mice was similar to MCAO+vehicle mice, without any statistical difference between the two groups. In the sham group, KC concentrations at 4 h, 7 and 24 h (data not shown) were significantly higher than in controls. Control mice: n = 7; sham mice: n = 5 to 8, except at 24 h, n = 3; MCAO mice: n = 6 to 10. *P <0.05, **P <0.01 indicate a significant difference between MCAO and sham mice. Results are presented as mean ± SEM. When placed over a data point, ∞, ⊗ and × indicate a significant .difference with concentrations in the same group at 0 h, 4 h and 7 h, respectively.

**Figure 2:**
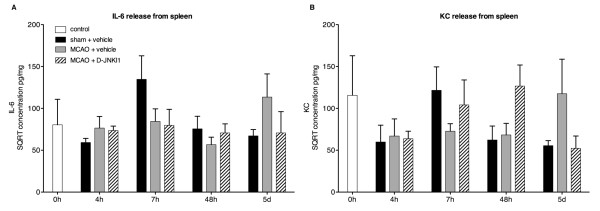
**Release of interleukin-6 and keratinocyte-derived chemokine from the spleen after middle cerebral artery occlusion.** Concentrations of IL-6 and KC were measured in the supernatants of spleen slice cultures and normalized to total protein concentrations. The graphs show the square root (SQRT) of (**A**) IL-6 and (**B**) KC concentrations in pg/mg at different time points from control (n = 7), sham (n = 4 to 8), MCAO+vehicle (n = 6 to 10) and MCAO+D-JNKI1 (n = 5 to 8) mice. IL-6 release from the spleen increased slightly, though not significantly, at 4 h in both MCAO+vehicle and MCAO+D-JNKI1 mice. Overall, there is no significant change in IL-6 and KC in spleen supernatants in any of the four groups tested. Results are represented as mean ± SEM.

The plasma concentrations of both IL-6 and KC increased markedly 4 h and 7 h after MCAO onset in vehicle-treated mice (Figure 1A,B; *P* <0.05). From 24 h (data not shown) to 48 h the concentrations decreased to control levels. Interestingly, as late as 5 d after ischemia, the secretion of IL-6 and KC increased again compared to shams (*P* = 0.054 and *P* <0.05, respectively). D-JNKI1 induced a non-significant reduction in IL-6 plasma levels at 4 h and 5 d (Figure 1A) while KC concentrations were not influenced by the treatment (Figure 1B). The 24 h time point was not tested in MCAO+D-JNKI1 mice as no significant difference was found between sham and MCAO+vehicle (not shown). Overall, we did not find a significant difference between MCAO+D-JNKI1 and MCAO+vehicle at any time points tested for either of the inflammatory mediators, despite a significant lesion volume reduction 48 h after MCAO with D-JNKI1 (36.4 ± 4.3 mm^3^ in vehicle mice, n *=* 7 and 19.0 ± 5.5 mm^3^ in D-JNKI1-treated mice, n *=* 5; *t*-test: *P* <0.05). In sham mice, the systemic IL-6 release did not differ from controls at any time point but there was a significant increase of KC at 4 h, 7 h and 24 h. While these findings suggest an early transient KC secretion into the plasma of sham mice due to the anesthesia [19] and the surgical procedure, the release of KC after MCAO was far greater. We also investigated the secretion of IL-6 and KC from the spleen at 4 h, 7 h, 48 h and 5 d in sham mice, control mice and after MCAO in mice with and without DJNKI1. We did not find any significant changes in either IL-6 or KC in spleen supernatants in any of the four groups tested (Figure 2).

**Figure 3:**
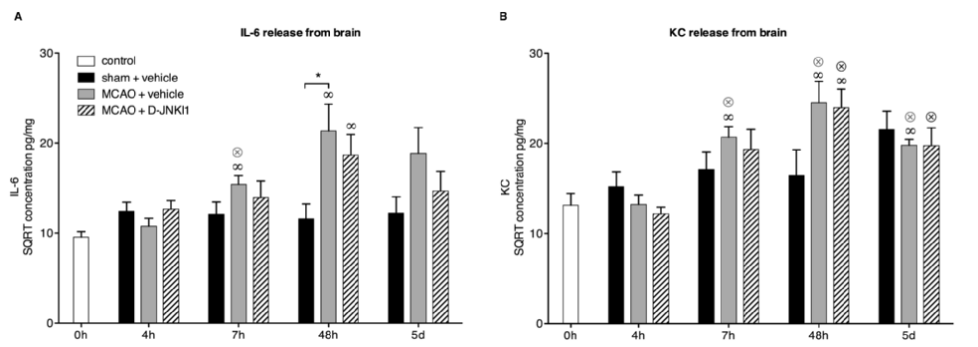
**Release of interleukin-6 and keratinocyte-derived chemokine from the brain after middle cerebral artery occlusion.** Concentrations of IL-6 and KC were measured in the supernatants of brain slice cultures and normalized to total protein concentrations. The graphs show the square root (SQRT) of IL-6 and KC concentrations in pg/mg at different time points in control, sham and MCAO mice. In sham mice, the cerebral release of IL-6 and KC did not differ from control mice at any time point. (**A**) Secretion of IL-6 from the brain tended to increase at 24 h (sham versus MCAO+vehicle: *P* = 0.06, data not shown) with a significant difference between sham and MCAO+vehicle 48 h after the occlusion. A slight, non-significant reduction of IL-6 release from the brain in mice treated with D-JNKI1 was seen at 5 d (MCAO+vehicle versus MCAO+D-JNKI1: *P* = 0.25). There was no significant difference of brain IL-6 release in D-JNKI1- and vehicle-treated mice at any time point. In MCAO + D-JNKI1 mice, IL-6 concentrations at 48 h remained significantly higher than in controls. (**B**) KC release in MCAO + vehicle mice started to increase significantly at 7 h compared to early time points (0 h and 4 h) and remained elevated at late time points. The brain KC release increased slightly, though not significantly, at 24 h (*P* = 0.19, data not shown) and 48 h (*P* = 0.08) after MCAO compared with sham mice. KC concentrations in MCAO + D-JNKI1 mice followed the same temporal profile as in MCAO + vehicle mice. Control mice: n = 7; sham mice: n = 5 to 8, except at 24 h, n = 3; MCAO mice: n = 6 to 10. *P <0.05 indicates a significant difference in MCAO mice and sham mice. Results are represented as mean ± SEM. When placed over a data point, ∞ and ⊗ indicate a significant difference with concentrations in the same group at 0 h and 4 h, respectively.

**Figure 4:**
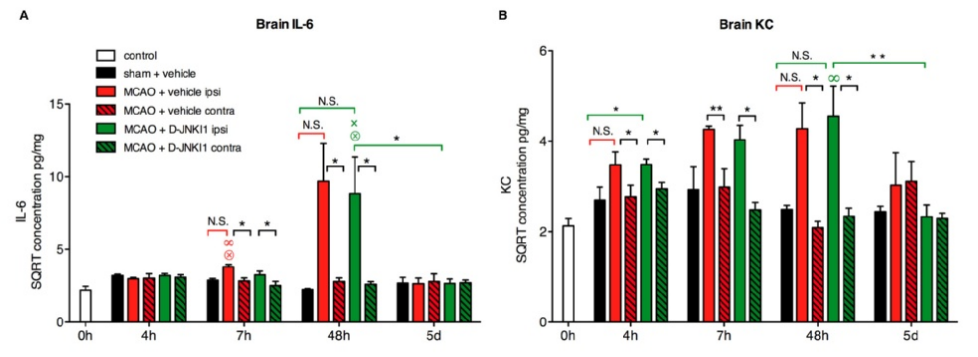
I**nterleukin-6 and keratinocyte-derived chemokine concentrations in brain homogenates.** Concentrations of IL-6 and KC were measured in brain homogenates and normalized to total protein concentrations. The graphs show the square root (SQRT) of IL-6 and KC concentrations in pg/mg at several time points from control, sham and MCAO mice. In sham mice, brain IL-6 and KC did not differ from control mice at any time points. (**A**) Brain IL-6 concentration in the hemisphere ipsilateral to the lesion in MCAO+vehicle mice increased at 7 h compared with control mice (0 h) and with the 4 h time point. A non-significant increase of brain IL-6 in MCAO+vehicle and MCAO+D-JNKI1 mice was seen at 48 h (MCAO+vehicle: *P* = 0.25; MCAO+D-JNKI1: *P* = 0.32; plain columns). There was no significant difference in brain IL-6 release between vehicle- and D-JNKI1-treated mice at any time points. In MCAO+D-JNKI1 mice, IL-6 concentrations at 48 h remained significantly higher than at 4 h and 7 h and decreased significantly at 5 d (*P* = 0.02). (**B**) Brain KC concentration in the ipsilateral hemisphere of MCAO mice increased slightly at 4 h in both groups (MCAO+vehicle: *P* = 0.08; MCAO+D-JNKI1: *P* = 0.04) and at 48 h (MCAO+vehicle: *P* = 0.27; MCAO+D-JNKI1: *P* = 0.17) compared with sham mice. KC concentrations in MCAO + D-JNKI1 mice at 48 h remained significantly higher than control mice and decreased significantly at 5 d (*P* = 0.006). Matched pairs analysis between the ipsilesional (ipsi, plain columns) and contralesional (contra, dashed columns) hemispheres showed that there is an increase in (**A**) brain IL-6 at 7 h and 48 h after MCAO in both groups and that (**B**) brain KC has already increased by 4 h and until 48 h in both MCAO groups compared with the contralesional hemisphere. Control mice: n = 3; sham mice: n = 3 to 5; MCAO + vehicle: n = 3 to 7; MCAO + D-JNKI1: n = 7 to 8. *P <0.05 and **P <0.02 indicates a significant difference. N.S.: not significant. Results are represented as mean ± SEM. When placed over a data point, ∞, ⊗ and × indicate a significant difference with concentrations in the same group at 0 h, 4 h and 7 h, respectively.

The release of IL-6 and KC from the brain increased significantly at 7 h compared to early time points (0 h and 4 h) and remained elevated at later time points after MCAO (Figure 3). In comparison with shams, there was an increase in IL-6 levels at 24 h (*P* = 0.06, not shown) and 48 h (*P* <0.05). A slight increase was also observed at 5 d, not reaching significance (*P* = 0.12; Figure 3A). Brain KC release tended to increase, although not significantly, at 24 h (*P* = 0.19, not shown) and 48 h (*P* = 0.08) after MCAO compared to shams (Figure 3B). The cerebral release of IL-6 and KC in sham mice did not differ from controls at any time point. A slight, non-significant reduction of brain IL-6 release was seen at 5 d with D-JNKI1 treatment. We did not find any significant change in brain IL-6 or KC release in D-JNKI1-treated mice at any time point, despite a significant lesion volume reduction at 48 h. To ensure that these results were not methodological artifacts, we also analyzed concentrations of IL-6 and KC in frozen brain sample homogenates from the same mice (Figure 4). We found a similar increase in brain IL-6 and KC 48 h after MCAO in both vehicle- and D-JNKI1-treated groups in the ipsilateral hemisphere compared with the contralateral (Figure 4), supporting our results of cytokine release from brain tissue (Figure 3).

**Figure 5:**
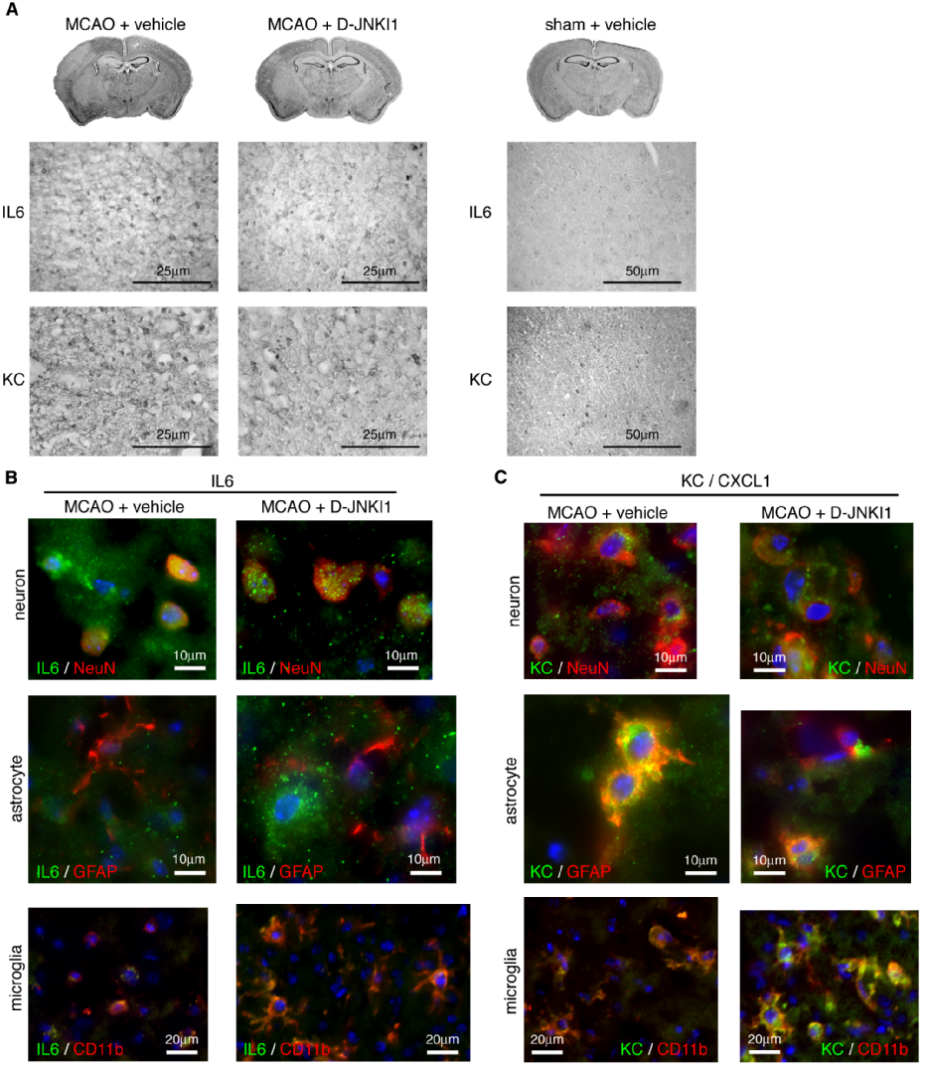
**Localization of interleukin-6 and keratinocyte-derived chemokine in the brain 48 h after middle cerebral artery occlusion.** Coronal brain sections were immunolabeled with specific antibodies 48 h after MCAO. (**A**) Representative IL-6 and KC immunohistochemistry of the ischemic cerebral cortex of vehicle- and D-JNKI1-treated animals (left panels). Both cytokines are up-regulated in the ischemic region compared with sham mice, which show little labeling (right panels). Nissl-stained coronal sections are shown at the top. The paler area is the ischemic lesion. (**B**, **C**) Double immunofluorescent staining of neurons (NeuN; red, top line), astrocytes (GFAP; red, middle line) or microglia (CD11b; red, bottom line), and IL-6 (green, left panel) or KC (green, right panel) in the ischemic cortex of vehicle- and D-JNKI1-treated mice. Yellow staining shows co-localization of IL-6 (B) with neurons and microglia but not astrocytes. KC (C) co-localizes mainly with astrocytes and microglia rather than neurons. D-JNKI1 treatment does not obviously change IL-6 and KC cell localization after MCAO.

To investigate the cellular localization of IL-6 and KC after MCAO, we performed immunohistochemistry in the cortex at 48 h, when we obtained significant neuroprotection with D-JNKI1. We found an up-regulation of IL-6 and KC in the infarcted tissue, in both vehicle-treated (n = 2 to 3) and D-JNKI1-treated (n = 2) mice compared with the contralateral hemisphere (data not shown) or to sham mice. Even though IL-6 and KC staining looks faint in the D-JNKI-treated mice, the labeling pattern and cellular localization were comparable between the two groups (Figure 5A). We performed double immunofluorescence using antibodies against IL-6, KC and NeuN, GFAP, CD11b to label neurons, astrocytes or microglial cells (peripheral macrophages and resident microglia), respectively. In MCAO-vehicle mice, IL-6 was found in neurons and microglia but not astrocytes, as previously described [20]. D-JNKI1-treated mice showed similar IL-6 labeling with slightly more ramified microglia [12] (Figure 5B). In both D-JNKI1- and vehicle-treated mice, KC was found predominantly in astrocytes and microglia, as previously described [21]. Neuronal KC labeling was non-nuclear. Overall, there was no difference in KC labeling between groups (Figure 5C).

In agreement with previously publications, we found an increase in IL-6 and KC in the blood of MCAO mice that precedes the release from the brain [15,22,23]. IL-6 and KC secretion from the spleen was not affected by MCAO. Furthermore, we showed, for the first time, a second, later systemic increase in these inflammatory mediators using two methods. The cellular location of IL-6 and KC in the brain was similar in both groups 48 h after ischemia. Importantly, our results show that neuroprotection mediated by D-JNKI1 after cerebral ischemia is independent of the early systemic release of IL-6 and KC, and reveal a later secretion from the brain.

## Discussion

After a stroke, cytokines and chemokines may play a pathogenic part in the lesion development, passively reflect the ischemic brain injury or have other physiological roles [2,20,24]. The increased production of IL-6 in patients with large strokes could be the result and/or the cause of the enlarged cerebral infarctions [20,25]. We hypothesized that the intravenous administration of neuroprotective agent D-JNKI1 may have anti-inflammatory properties that attenuate the lesion size. On the contrary, we demonstrated that the early systemic release of IL-6 and KC and later increase in the brain are not significantly reduced by D-JNKI1 treatment. Others have published that the related JNK inhibitor, L-JNKI1, did not reduce brain cytokine or chemokine mRNA expression 3 h after perinatal hypoxia–ischemia despite neuroprotection [26].

The results show that brain KC and IL-6 production, although regulated by JNK [16], is not affected by these JNK inhibitors after cerebral ischemia and suggest that neuroprotection through JNK inhibition occurs independently of IL-6 and KC in this model and at the time points investigated. Others showed that D-JNKI1 significantly prevented liver injury after hemorrhage and resuscitation by inhibiting cytokine production [13], which is also true in a model of uveitis [14]. This suggests that D-JNKI1 could have distinct anti-inflammatory effects depending on the disease, its severity, the time and route of administration and administered dose.

In our MCAO model, the early systemic increase in IL-6 and KC is linked to the cerebral arterial occlusion but may not necessarily predict an unfavorable outcome. It is possible, however, that these inflammatory mediators contribute to the damage observed in DJNKI1-treated mice. The source of the systemic cytokines remains to be defined. We found no significant change in splenic secretion, in contrast with severe ischemia [23], suggesting that moderate ischemia induces less systemic inflammation. In the ischemic brain at 48 h, neurons and microglia were the major source of IL-6 while KC originated mostly from glial cells, with and without D-JNKI1 treatment. Furthermore, our results suggest that the second systemic increase in inflammatory mediators in mice with MCAO reflects ongoing tissue damage, possibly influencing the outcome at later time points.

Although the overall survival of the mice in these experiments was not significantly different between groups (data not shown), no death was recorded beyond 24 h after ischemia in the D-JNKI1-treated group. D-JNKI1 may therefore have a delayed beneficial physiopathological effect after MCAO, explaining the slight systemic decrease in IL-6 and KC at 5 d in D-JNKI1-treated mice (Figures 3 and 4) but this will require further investigation.

In summary, we demonstrate that neuroprotection with D-JNKI1 does not significantly reduce brain and early systemic secretion of IL-6 and KC after moderate ischemia and that the spleen is not the major source of the early systemic secretion. Taken together with our earlier studies [12], these results show that neuroprotection by JNK inhibition occurs independently of microglial activation and IL-6 and KC secretion, an important observation in a widely used stroke model with striking neuroprotection. Brain-immune system interactions are complex in experimental ischemia models and detailed characterizations of the differential effects of the model on observed immune system effects are important.

## Abbreviations

ELISA, Enzyme-linked immunosorbent assay; IL-6, Interleukin-6; JNK, c-Jun NH2-terminal kinase; KC/CXCL1, Keratinocyte-derived chemokine; MCAO, Middle cerebral artery occlusion; PBS, Phosphate-buffered saline; SEM, Standard error of the mean.

## Competing interests

AV and CP were employed by Xigen SA, a company that has been involved in developing D-JNKI1. LH has acted as a consultant for Xigen SA.

## Authors’ contributions

CB designed the study, performed the experiments, analyzed the data, prepared the figures and drafted the manuscript. AV and CP designed the study, helped to analyze the data and draft the manuscript. LH designed and coordinated the study and helped to analyze the data and draft the manuscript. All authors read and approved the final manuscript.
